# Follow-up and Mortality Profiles in the Miyagi Cohort Study

**DOI:** 10.2188/jea.14.S2

**Published:** 2005-03-18

**Authors:** Ichiro Tsuji, Yoshikazu Nishino, Yoshitaka Tsubono, Yoshinori Suzuki, Atsushi Hozawa, Naoki Nakaya, Kazuki Fujita, Shinichi Kuriyama, Daisuke Shibuya, Akira Fukao, Shigeru Hisamichi

**Affiliations:** 1 Division of Epidemiology, Department of Public Health and Forensic Medicine, Tohoku University Graduate School of Medicine.; 2 Miyagi Cancer Society.; 3 Department of Public Health, Yamagata University School of Medicine.

**Keywords:** prospective cohort study, lifestyle, follow-up, mortality

## Abstract

BACKGROUND: Findings from a large-scale population-based prospective cohort would lead us to better understanding of the relationship between lifestyle and health, thus better provision of strategies for disease prevention and health promotion.

METHODS: We conducted a baseline survey with two self-administered questionnaires regarding lifestyle and personality on the residents aged 40 to 64 years in 14 municipalities of Miyagi Prefecture, Japan, during June through August, 1990. Out of the eligible 51,925 residents, 47,605 (91.7%) responded to the lifestyle questionnaire and formed the cohort under study. We then have been following up the subjects for mortality, migration, and incidence of cancer.

RESULTS: During the follow-up from June 1990 through March 2001, 2,536 subjects (5.3%) died and 2,166 subjects (4.5%) emigrated. The distribution of the causes of death among the study subjects was quite consistent with the national average.

CONCLUSIONS: In this cohort study, both the participation rate and the follow-up rate are satisfactorily high. We expect this Miyagi Cohort Study to provide the society with evidence for health promotion and disease prevention.

In order to investigate the effect of lifestyle upon health, we established a population-based cohort of the residents aged 40 to 64 years in 14 municipalities of Miyagi Prefecture, Japan, during June through August, 1990. Since the study design, response rate and baseline characteristics of the cohort subjects has been already reported in detail,^1^ we here would summarize these issues.

We randomly selected 14 out of 62 towns or villages at Miyagi Prefecture as a study area. We asked these 14 municipal offices to provide residential registry of the above age range as of 1 April, 1990. Of 53,464 subjects (26,460 men and 27,004 women) at registry, we excluded persons who are hospitalized or away on business for extended length of time. Then, 51,921 subjects (25,279 men and 26,642 women) were eligible for the baseline survey. From June to August 1990, the leaders of public health activities in each municipality visited the subjects to deliver a questionnaire, and asked them to complete it. The same personnel revisited the subjects to collect the questionnaire a few weeks later. The self-administered questionnaire was consisted of two parts. The first was a lifestyle questionnaire which consisted of about 90 items concerning 12 factors (past medical history, family history, recent physical conditions, drinking habit, smoking habit, dietary habit, occupation, marital status, education, screening history, health insurance, and reproductive history for women). The second questionnaire was a Japanese translation of the original English version of the short-form Eysenck Personality Questionnaire-Revised, one of a series of personality inventories developed by Eysenck and colleagues.^2^

Out of the eligible 51,921 residents, 47,605 (91.7%) responded to the first questionnaire and formed the cohort under study. The subjects, then, have been followed up for mortality, migration, and incidence of cancer.

In this article, we would report follow-up status and mortality profile in this cohort study between June 1990 and March 2001.

## METHODS

In order to follow up the subjects for migration and mortality, we established the Follow-up Committee. The Committee was consisted of Miyagi Cancer Society; Community Health Division of all 14 municipalities; Department of Health and Welfare, Miyagi Prefectural Government; and Division of Epidemiology, Tohoku University Graduate School of Medicine. The Committee periodically reviewed the Residential Registration Record of each municipality. It is generally accepted that this registration system in Japan is accurate and reliable because this is the basis for tax, pension, and human services provided by the municipalities. So far, the Committee reviewed the Record five times (Table 1).

**Table 1.** Review process of residential registration record.

	Time of review	Period reviewed
First	July-August 1992	1990.6.1∼1992.3.31
Second	April-September 1995	1992.4.1∼1995.3.31
Third	July-September 1997	1995.4.1∼1997.3.31
Fourth	July-October 1999	1997.4.1∼1999.3.31
Fifth	July-October 2001	1999.4.1∼2001.3.31

With this review, we identified the subjects who either died or emigrated during observation. For both decedents and emigrants, we recorded the date of death/emigration. For decedents, we investigated cause of death by reviewing the death certificates of the subjects at Public Health Centers of the study area. The underlying cause of death was coded according to International Classification of Diseases, the Ninth Revision (ICD-9).^3^ For emigrants, we obtained no information regarding a new address. Therefore, the emigrants were lost to follow-up at the time of emigration.

We calculated the mortality rate among the study subjects for all causes, cancer, heart disease, and cerebrovascular disease, respectively. Mortality rate was calculated as the number of decedents divided by the number of person-years of follow-up. In classifying causes of death, we defined cancer as ICD-9 Codes from 140 through 208; heart disease as ICD-9 Codes from 393 through 398 and those from 410 through 429; and cerebrovascular disease as ICD-9 Codes from 430 through 438.

## RESULTS

Table 2 indicates the number of decedents and emigrants by sex and five-year age group. Out of 47,605 cohort subjects, 2,536 subjects (5.3%) died and 2,166 subjects (4.5%) emigrated. The death rates increased with age, and were larger in men than in women.

**Table 2.** The number of decedents and emigrants by 5-year age category.

	Age at baseline (year)					Total

	40-44	45-49	50-54	55-59	60-64	
Men						
Alive	5,160 (90.7)	3,357 (90.8)	3,540 (90.1)	4,025 (86.4)	4,025 (82.7)	20,107 (88.0)
Dead	122 (2.1)	160 (4.3)	234 (6.0)	478 (10.3)	707 (14.5)	1,701 (7.4)
Emigrated	407 (7.2)	179 (4.8)	154 (3.9)	154 (3.3)	134 (2.8)	1,028 (4.5)
Total	5,689 (100)	3,696 (100)	3,928 (100)	4,657 (100)	4,866 (100)	22,836 (100)

Women	4,895 (92.3)	3,637 (92.4)	4,273 (93.8)	4,932 (91.7)	5,059 (90.3)	22,796 (92.0)
Alive	66 (1.3)	84 (2.1)	104 (2.3)	220 (4.1)	361 (6.5)	835 (3.4)
Dead	340 (6.4)	214 (5.4)	179 (3.9)	224 (4.2)	181 (3.2)	1,138 (4.6)
Emigrated	5,301 (100)	3,935 (100)	4,556 (100)	5,376 (100)	5,601 (100)	24,769 (100)
Total						

Percentages in parentheses.

Table 3 indicates the person-years of follow-up, the number of decedents and mortality for all causes, cancer, heart disease, cerebrovascular disease, respectively, by sex and five-year age group. For both men and women, the most common cause of death was cancer (n=1,132), occupying 43.6% of all deaths in men and 46.1% in women. Deaths from heart disease (n=358) occupied 15.2% in men and 11.9% in women. Deaths from cerebrovascular disease (n=307) occupied 11.1% in men and 14.3% in women. The distribution of the causes of death among the study subjects was quite consistent with the national average. In 1995, among the decedents aged 40 to 64 years in Japan, cancer occupied 40.1% in men and 48.2% in women, heart disease occupied 12.3% in men and 9.3% in women, and cerebrovascular disease occupied 10.3% in men and 11.2% in women.^4^

**Table 3.** The number of decedents and mortality rates^†^ for specific causes by 5-year age category.

	Age at baseline (year)					Total

	40-44	45-49	50-54	55-59	60-64	
Men						
Person-years	58,678	38,277	40,581	47,283	48,739	233,558
All causes						
n	122 (100)	160 (100)	234 (100)	478 (100)	707 (100)	1,701 (100)
Mortality	207.9	418.0	576.6	1010.9	1450.6	728.3
Cancer						
n	31 (25.4)	64 (40.0)	107 (45.7)	211 (44.1)	329 (46.5)	742 (43.6)
Mortality	52.8	167.2	263.7	446.2	675.0	317.7
Heart disease						
n	16 (13.1)	19 (11.9)	33 (14.1)	77 (16.1)	114 (16.1)	259 (15.2)
Mortality	27.3	49.6	81.3	162.8	233.9	110.9
Cerebrovascular disease						
n (%)	17 (13.9)	20 (12.5)	22 (9.4)	47 (9.8)	82 (11.6)	188 (11.1)
Mortality	29.0	52.3	54.2	99.4	168.2	80.5

Women						
Person-years	55,142	40,955	47,798	56,046	58,017	257,958
All causes						
n	66 (100)	84 (100)	104 (100)	220 (100)	361 (100)	835 (100.0)
Mortality	119.7	205.1	217.6	392.5	622.2	323.7
Cancer						
n	27 (40.9)	34 (40.5)	52 (50.0)	113 (51.4)	164 (45.4)	390 (46.7)
Mortality	49.0	83.0	108.8	201.6	282.7	151.2
Heart disease						
n	12 (18.2)	14 (16.7)	12 (11.5)	18 (8.2)	43 (11.9)	99 (11.9)
Mortality	21.8	34.2	25.1	32.1	74.1	38.4
Cerebrovascular disease						
n	8 (12.1)	11 (13.1)	10 (9.6)	31 (14.1)	59 (16.3)	119 (14.3)
Mortality	14.5	26.9	20.9	55.3	101.7	46.1

† : per 100,000 person-years

Percentages in parentheses.

Table 4 indicates the number of decedents and mortality for varying sites of cancer by sex and five-year age. In men, the most common cause of cancer deaths was stomach (n=150; 20.2% of all cancer deaths), followed by lung (n=138; 18.6%), colorectum (n=81; 10.9%), and liver (n=74; 10.0%). In women, stomach (n=55; 14.1%) was the most common, followed by colorectum (n=49; 12.6%), breast (n=46; 11.8%), and pancreas (n=45; 11.5%).

**Table 4.** The number of decednets and mortality rates^†^ for site-specific cancer by 5-year age category.

		Age at baseline (year)											Total	

		40-44		45-49		50-54		55-59			60-64			
Men														

Esophagus	n	1	(3.2)	4	(6.3)	6	(5.6)	19		(9.0)	29	(8.8)	59	(8.0)
	Mortality		1.7		10.5		14.8			40.2		59.5		25.3
Stomach	n	6	(19.4)	19	(29.7)	23	(21.5)	38		(18.0)	64	(19.5)	150	(20.2)
	Mortality		10.2		49.6		56.7			80.4		131.3		64.2
Colon	n	1	(3.2)	5	(7.8)	4	(3.7)	13		(6.2)	17	(5.2)	40	(5.4)
	Mortality		1.7		13.1		9.9			27.5		34.9		17.1
Rectum	n	5	(16.1)	4	(6.3)	6	(5.6)	12		(5.7)	14	(4.3)	41	(5.5)
	Mortality		8.5		10.5		14.8			25.4		28.7		17.6
Liver	n	3	(9.7)	5	(7.8)	14	(13.1)	23		(10.9)	29	(8.8)	74	(10.0)
	Mortality		5.1		13.1		34.5			48.6		59.5		31.7
Biliary tract	n	2	(6.5)	2	(3.1)	3	(2.8)	7		(3.3)	16	(4.9)	30	(4.0)
	Mortality		3.4		5.2		7.4			14.8		32.8		12.8
Pancreas	n	3	(9.7)	4	(6.3)	9	(8.4)	20		(9.5)	22	(6.7)	58	(7.8)
	Mortality		5.1		10.5		22.2			42.3		45.1		24.8
Lung	n	4	(12.9)	9	(14.1)	19	(17.8)	31		(14.7)	75	(22.8)	138	(18.6)
	Mortality		6.8		23.5		46.8			65.6		153.9		59.1
Prostate	n	1	(3.2)	1	(1.6)	2	(1.9)	8		(3.8)	5	(1.5)	17	(2.3)
	Mortality		1.7		2.6		4.9			16.9		10.3		7.3
Leukemia	n	1	(3.2)	2	(3.1)	3	(2.8)	4		(1.9)	8	(2.4)	18	(2.4)
	Mortality		1.7		5.2		7.4			8.5		16.4		7.7
Women														

Esophagus	n	0	(0.0)	0	(0.0)	1	(1.9)	1		(0.9)	2	(1.2)	4	(1.0)
	Mortality		0.0		0.0		2.1			1.8		3.4		1.6
Stomach	n	7	(25.9)	7	(20.6)	11	(21.2)	13		(11.5)	17	(10.4)	55	(14.1)
	Mortality		12.7		17.1		23.0			23.2		29.3		21.3
Colon	n	2	(7.4)	2	(5.9)	4	(7.7)	6		(5.3)	12	(7.3)	26	(6.7)
	Mortality		3.6		4.9		8.4			10.7		20.7		10.1
Rectum	n	0	(0.0)	2	(5.9)	5	(9.6)	7		(6.2)	9	(5.5)	23	(5.9)
	Mortality		0.0		4.9		10.5			12.5		15.5		8.9
Liver	n	0	(0.0)	0	(0.0)	0	(0.0)	6		(5.3)	11	(6.7)	17	(4.4)
	Mortality		0.0		0.0		0.0			10.7		19.0		6.6
Biliary tract	n	1	(3.7)	0	(0.0)	2	(3.9)	6		(5.3)	15	(9.2)	24	(6.2)
	Mortality		1.8		0.0		4.2			10.7		25.9		9.3
Pancreas	n	0	(0.0)	4	(11.8)	3	(5.8)	15		(13.3)	23	(14.0)	45	(11.5)
	Mortality		0.0		9.8		6.3			26.8		39.6		17.4
Lung	n	2	(7.4)	5	(14.7)	5	(9.6)	12		(10.6)	18	(11.0)	42	(10.8)
	Mortality		3.6		12.2		10.5			21.4		31.0		16.3
Breast	n	8	(29.6)	5	(14.7)	8	(15.4)	13		(11.5)	12	(7.3)	46	(11.8)
	Mortality		14.5		12.2		16.7			23.2		20.7		17.8
Uterus	n	0	(0.0)	4	(11.8)	3	(5.8)	5		(4.4)	3	(1.8)	15	(3.8)
	Mortality		0.0		9.8		6.3			8.9		5.2		5.8
Ovary	n	1	(3.7)	1	(2.9)	2	(3.9)	7		(6.2)	5	(3.1)	16	(4.1)
	Mortality		1.8		2.4		4.2			12.5		8.6		6.2
Leukemia	n	2	(7.4)	0	(0.0)	1	(1.9)	2		(1.8)	2	(1.2)	7	(1.8)
	Mortality		3.6		0.0		2.1			3.6		3.4		2.7

† : per 100,000 person-years

Percentages in parentheses.

## DISCUSSION

In this article, we reported the method of following-up the cohort subjects and the number of decedents and emigrants during the follow-up from June 1990 through March 2001 in the Miyagi Cohort Study. Although we stopped following the emigrants at the time when they moved, the emigration rate was as low as 4.5%. We have documented 2,536 deaths, which seemed to be large enough for us to analyze the association between the health-related lifestyle and the risk for mortality. With this dataset, we would examine and report the effect of lifestyle such as smoking, alcohol drinking, body mass index, physical activity, and so forth in this Supplement.

We would further continue to follow up the cohort subjects, and we expect this Miyagi Cohort Study to provide the society with evidence for health promotion and disease prevention.
